# Population pharmacokinetics and exposure–response relationship of the antituberculosis drug BTZ-043

**DOI:** 10.1093/jac/dkaf076

**Published:** 2025-03-28

**Authors:** Simon E Koele, Norbert Heinrich, Veronique R De Jager, Julia Dreisbach, Patrick P J Phillips, Petra Gross-Demel, Rodney Dawson, Kim Narunsky, Leticia M Wildner, Timothy D Mchugh, Lindsey H M Te Brake, Andreas H Diacon, Rob E Aarnoutse, Michael Hoelscher, Elin M Svensson

**Affiliations:** Department of Pharmacy, Radboud Institute for Medical Innovation, Radboud University Medical Center, Nijmegen, The Netherlands; Institute of Infectious Diseases and Tropical Medicine, LMU University Hospital, LMU, Munich, Germany; German Center for Infection Research (DZIF), Munich Partner Site, Munich, Germany; Fraunhofer Institute for Translational Medicine and Pharmacology ITMP, Immunology, Infection and Pandemic Research, Munich, Germany; TASK, Cape Town, South Africa; Institute of Infectious Diseases and Tropical Medicine, LMU University Hospital, LMU, Munich, Germany; German Center for Infection Research (DZIF), Munich Partner Site, Munich, Germany; UCSF Center for Tuberculosis, University of California San Francisco, San Francisco, USA; Institute of Infectious Diseases and Tropical Medicine, LMU University Hospital, LMU, Munich, Germany; German Center for Infection Research (DZIF), Munich Partner Site, Munich, Germany; Department of Medicine, University of Cape Town Lung Institute, Cape Town, South Africa; Department of Medicine, University of Cape Town Lung Institute, Cape Town, South Africa; UCL Centre for Clinical Microbiology, University College London, London, UK; UCL Centre for Clinical Microbiology, University College London, London, UK; Department of Pharmacy, Radboud Institute for Medical Innovation, Radboud University Medical Center, Nijmegen, The Netherlands; TASK, Cape Town, South Africa; Department of Pharmacy, Radboud Institute for Medical Innovation, Radboud University Medical Center, Nijmegen, The Netherlands; Institute of Infectious Diseases and Tropical Medicine, LMU University Hospital, LMU, Munich, Germany; German Center for Infection Research (DZIF), Munich Partner Site, Munich, Germany; Fraunhofer Institute for Translational Medicine and Pharmacology ITMP, Immunology, Infection and Pandemic Research, Munich, Germany; Unit Global Health, Helmholtz Zentrum München, German Research Center for Environmental Health (HMGU), Neuherberg, Germany; Department of Pharmacy, Radboud Institute for Medical Innovation, Radboud University Medical Center, Nijmegen, The Netherlands; Department of Pharmacy, Uppsala University, Uppsala, Sweden

## Abstract

**Introduction:**

BTZ-043 is a first-in-class benzothiazinone for the treatment of TB with demonstrated early bactericidal activity. BTZ-043 is metabolized into two major metabolites: M1 and M2. The aim of this study was to characterize the pharmacokinetics (PK) and early exposure–response (pharmacokinetic/pharmacodynamic, PK/PD) relationship for BTZ-043.

**Methods:**

A population PK/PD model for BTZ-043 and its metabolites was developed using data from a sequential Phase 1b/2a, randomized, controlled clinical trial in participants with pulmonary TB. BTZ-043 was administered in daily doses ranging from 250 to 1750 mg over 14 days. The decrease in bacterial load was determined by culture of sputum samples to quantify cfu on solid medium, and time to positivity in liquid medium.

**Results:**

In total, 77 participants received the experimental treatment. PK were best described by two-compartment disposition models for BTZ-043 and M2, and a one-compartment disposition model for M1. When given without food, the bioavailability was 54% (95% CI: 43%–65%) lower than with food. The decrease in bacterial load was described by a bilinear model with estimated node at 48 h. Participants in the highest dose group in Stage 2 (1000 mg) had a 2-fold faster decrease in mycobacterial load during the initial 2 days compared with participants in the lowest dose group (250 mg), driven by an E_max_ relationship to the BTZ-043_total_ exposure (BTZ-043 + M2).

**Conclusions:**

We characterized the population PK/PD of BTZ-043 in trial participants with pulmonary TB. An exposure–response relationship was only apparent for the first 2 days on treatment, indicating the need for further dose-finding studies.

## Introduction

TB remains the deadliest infectious disease in the 21st century. There is an urgent need for novel therapeutic options, considering the lengthy treatment duration, prevalent adverse effects, and development of resistance to recently approved drugs.^1^

BTZ-043 is a novel first-in-class benzothiazinone for the treatment of TB.^2,3^ It covalently binds to the DprE1 enzyme, inhibiting cell wall biosynthesis.^2^*In vitro* analyses showed that BTZ-043 is active against drug-susceptible and MDR clinical isolates.^4^ Strong BTZ-043 dose-dependent killing was observed in a mouse TB model.^5^ Furthermore, early bactericidal activity (EBA) was demonstrated in a combined Phase 1b/2a study with an effect over 14 days (−0.100 to −0.115 log_10_ cfu/mL/day) similar to the standard dose (10 mg/kg daily) of rifampicin (−0.093 log_10_ cfu/mL/day).^6^ BTZ-043 is generally well tolerated with mild to moderate adverse effects.^6^

The pharmacokinetics (PK) of BTZ-043 are characterized by a relatively short half-life (1–2 h), with a double peak observed at higher doses. BTZ-043 is partly metabolized in the liver into the amino derivate M1, demonstrated in liver microsome preparations. M1 has a longer half-life compared with the parent (8–9 h). However, the majority of BTZ-043 is present in the form of M2, a so-called Meisenheimer complex, with peak concentrations 3–5-fold higher compared with the parent. M2 is unstable in the presence of atmospheric oxygen and tends to convert back into BTZ-043.^3^ Several other pathways have been identified but are thought to only have a minor contribution to metabolism in humans.

*In vitro* experiments with BTZ-043 have shown high activity against strains of *Mycobacterium tuberculosis*.^5,7^ M1 activity was substantially lower compared with BTZ-043 activity. As M2 is unstable *in vitro* and tends to convert back into the parent, it is unknown if M2 contributes to the antimicrobial effect of BTZ-043 or if this is driven by the unchanged parent.

To characterize the PK and the early exposure–response relationship for BTZ-043, we developed a non-linear mixed-effects population pharmacokinetic/pharmacodynamic (PK/PD) model of BTZ-043 and its major metabolites.

## Methods

### Ethics

All trial procedures were approved by the local ethical authorities and compliant with good clinical practice guideline and the Declaration of Helsinki (SAHPRA; Ref 20190606).

### Clinical study

Data were obtained from a sequential Phase 1b/2a randomized dose-expansion clinical study that aimed to evaluate the safety, tolerability and EBA of BTZ-043 (clinicaltrials.gov id: NCT04044001). Participants aged between 18 and 64 years with drug-susceptible TB were eligible for inclusion. The trial procedures and main results were previously described in detail.^6^

In summary, the study was performed in two stages. In Stage 1 (Phase 1b), participants received orally administered daily doses of BTZ-043 for 14 days with dose escalation after each cohort. Dose escalation was informed using an adaptive continual reassessment method.^8^ Doses administered ranged between 250 and 1750 mg daily. Three participants were included in each dosing group and six participants were included in the highest dosing group. An interim analysis determined the doses to be administered in the second stage. In this Stage 2 (Phase 2a), 54 participants were randomized into four groups, to receive either BTZ-043 in doses of 250, 500 or 1000 mg daily or the control regimen with standard doses of the first-line TB drugs isoniazid, rifampicin, pyrazinamide and ethambutol (administered as Rifafour e-275^®^) for 14 days in a 3:3:3:2 ratio.

To evaluate the effects of food on the PK, participants received BTZ-043 in a fasted state in Stage 1 from Day 1 to Day 12, and with a high-fat breakfast on Day 14. In Stage 2, participants took BTZ-043 either prior to or 30 min after the start of intake of a standard breakfast.

### PK analysis

Intensive plasma PK sampling was performed on Days 1, 12 and 14 for Stage 1 and on Days 1 and 14 for Stage 2. During the intensive PK sampling days, plasma samples were drawn at 0, 0.25, 0.5, 1, 1.5, 2, 3, 4, 6, 8, 10, 12 and 24 h after the dose.

Plasma concentrations of BTZ-043, M1 and BTZ-043_total_ (BTZ-043 + M2) were quantified for all sampling timepoints using validated LC-MS-MS assays by GLP-accredited bioanalytical laboratory Nuvisan (Neu Ulm, Germany). Two assays were used as the M2 metabolite cannot be directly quantified. The first assay converted all available M2 back into BTZ-043 under acid conditions and quantified the BTZ-043_total_ (i.e. BTZ-043 + M2), and the second assay quantified the BTZ-043 and M1 metabolite. M2 concentrations were subsequently determined by subtracting the BTZ-043 concentration from the BTZ-043_total_ concentration. The lower limit of quantification was determined as 20 ng/mL for all analytes. More details regarding the bioanalysis are provided in the Supplementary materials (available as Supplementary data at *JAC* Online).

### PK model development

One- two- and three-compartment disposition models for each analyte were evaluated. Concentration data were transformed to molar units to preserve mass balance. PK parameters for BTZ-043, M1 and M2 were estimated simultaneously to account for the interdependence of their PK. To keep the model parameters identifiable, the bioavailability and fractions metabolized per path were set to 1, rendering disposition parameters relative to the true values. Clearance was assumed to take place from the central plasma compartments. Linear and non-linear elimination for each analyte were investigated. First- and zero-order absorption models were tested, as well as absorption through transit compartments. Parallel absorption was tested, using first-order absorption with or without a lag time.

Inter-individual variability (IIV) was assumed to be log-normally distributed and explored on all structural parameters included in the final model. Each PK sampling day was treated as a separate occasion to specify the inter-occasion variability (IOV). Additive, proportional and combined additive and proportional residual error models were explored. All volumes and clearances were normalized to 70 kg and allometrically scaled, using fixed exponents of 1 and 0.75, respectively. Covariates investigated included age, food, time and self-identified race on clearances, sex on volumes, and food type on the bioavailability and absorption parameters. Covariates were included based on scientific plausibility, clinical relevance of the effect, and statistical significance.

M2 is unstable in frozen plasma and therefore BTZ-043 levels increase over time by back-conversion of M2 to BTZ-043, even in −80°C storage. For this reason, analysis of BTZ-043 needs to be completed within 56 days after sample collection, which is not feasible in many settings. In contrast, BTZ-043_total_ and M1 are stable for at least 6 months, making them preferable for future studies. To assess whether these analytes could be used as reliable surrogates in future studies, the AUC_0–24_ values of all analytes at Day 12 for Stage 1 and Day 14 for Stage 2 was determined using the final PK model, with only M1 and BTZ-043_total_ concentrations as input for the PK model. These exposure metrics were subsequently compared using linear regression to the AUC_0–24_ values when determined using BTZ-043, M1 and M2 concentrations as input.

### Bacterial load quantification

Overnight sputum samples were collected before the start of treatment and on Days 2, 3, 4, 6, 8, 11 and 14 of treatment. All sputum samples were cultured to determine the cfu on solid medium, and the time to positivity (TTP) in the MGIT system, as previously described.^9^ For all analyses in this paper, we consider cfu observations lower than 1.0 cfu/mL and TTP observations over 25 days as a negative culture result, respectively, since this resulted in better model fit, as has now been shown in other trials.^10^

### PD model development

Log_10_-transformed cfu and TTP data were simultaneously analysed to describe the decrease in bacterial load. Linear, bilinear, smooth bilinear and semimechanistical models were considered to describe the cfu and TTP over time.^11,12^ Correlations between baseline bacterial load and killing parameters connected the cfu and TTP models. IIV was assumed to be log-normally distributed and explored on all structural parameters.

The so-called NONMEM ‘M3’ method, incorporating a partial likelihood, was used to account for censored observations.^13^ A probability for obtaining a negative culture at baseline was included for cfu and TTP based on the average chance of obtaining a negative culture result during the first 2 days of treatment, reflecting the sensitivity of the bacterial load quantification.

Stepwise covariate modelling (SCM) was performed. Covariates explored were age, weight, study site and baseline cfu and TTP. A forward inclusion significance criterion of 0.05 and a backward elimination significance criterion of 0.01 was used. The final model identified by the SCM was taken forward to evaluate the exposure–response relationship.^14^

The exposure–response relationship of BTZ-043 was investigated using individual model-derived estimates of the AUC_0–24_ and *C*_max_ during the intensive PK sampling on Day 12 for Stage 1 and Day 14 for Stage 2. Linear and E_max_ exposure–response models were explored with BTZ-043, M1, M2 and BTZ-04_total_ (BTZ-043 + M2). The exposure–response relationship was evaluated over the entire trial duration, or by estimating separate effects for different time intervals.

### Model evaluation

PK and PD modelling was performed with NONMEM 7.5 using Pirana 2.9.9 as graphical interface and Perl-speaks-NONMEM 5.3.0 for additional functionalities.^15–17^ Dataset preparation, data management, plot generation and linear regression analysis was performed in R 4.1.3 using R Studio 2022.02.^18,19^ Graphical evaluation of the PK and PD models was performed with goodness-of-fit plots, and visual predictive checks (VPCs). A decrease in objective function value (dOFV) of −3.84 was considered statistically significant, corresponding to a *P* value of 0.05. Parameter precision for the PK and PD models was determined using the sampling importance resampling (SIR) method.^20^

## Results

A total of 24 and 45 participants were enrolled on a BTZ-043-containing arm in Stages 1 and 2 of the trial, respectively. We included 24 participants from Phase 1 and 44 participants from Stage 2 in the analysis. No data were available for one participant in Stage 2 (500 mg dosing group) due to withdrawal before the study procedures. A summary of participant demographics is presented in Table 1.

**Table 1.** Demographic information of participants included in Stage 1 and in Stage 2, randomized to receive an oral administration of BTZ-043

Variable (unit)	Stage 1	Stage 2	Combined Stage 1 + 2
Total participants, *n*	24	44	68
Age (years), median (range)	26 (20–57)	27 (18–56)	27 (18–57)
Weight (kg), median (range)	55 (47–81)	53 (42–71)	54 (42–81)
Height (m), median (range)	1.7 (1.5–1.9)	1.7 (1.6–1.9)	1.7 (1.5–1.9)
Male sex, *n* (%)	17 (71)	40 (90)	57 (84)
HIV-1 negative, *n* (%)	24 (100)	44 (100)	68 (100)
Self-identified race, *n* (%)			
Black	15 (63)	29 (66)	44 (65)
Cape-coloured	8 (33)	15 (34)	23 (34)
White	1 (4)	0 (0)	1 (1)
Food^a^, *n* (%)			
Fasted	24 (100)	0 (0)	24
High-fat	19 (100)	0 (0)	19
Standard together with dose	0 (0)	24	24
Standard 30 min prior to dose	0 (0)	20	20

^a^Participants in Stage 1 had BTZ-043 while fasting on Days 1–11 and with a high-fat meal on Day 14.

### PK model

There were a total of 1808 PK observations for BTZ-043, 1808 for M1 and 1793 for M2 available for analysis. In total, 600 (33%) observations for BTZ-043, 159 (8.8%) for M1 and 146 (8.1%) for M2 were below the limit of quantification (BLQ). PK models utilizing the M3 method to account for BLQ observations were highly unstable, and not suitable for log-likelihood ratio testing. Therefore, BLQ observations were excluded from the analysis. The final model, developed without the M3 method, was re-estimated with the M3 method to assess potential model misspecification. This model described the proportion of BLQ observations better than the model with all BLQ data excluded, but significantly underestimated the central tendency of the BTZ-043 and M2 elimination phases. Additionally, the model did not minimize properly (common with M3 methods) and the change in parameter estimates was limited.^21^

A two-compartmental disposition model provided the best model fit for BTZ-043 and M2, and a one-compartmental disposition model for M1 was identified. Absorption of BTZ-043 was characterized by two transit compartments, followed by a first-order absorption into the BTZ-043 central compartment through an absorption compartment.

**Figure 1.**
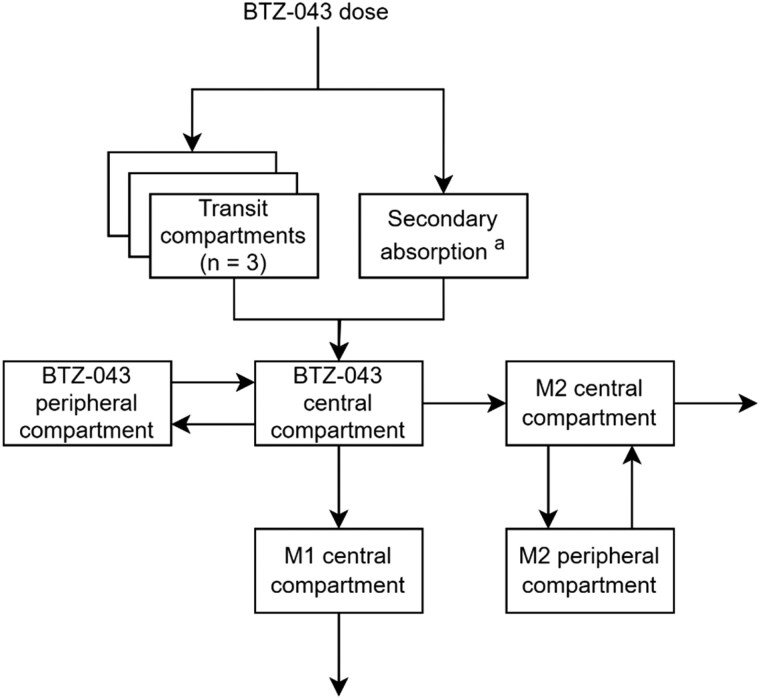
Schematic representation of the structural PK model. ^a^Secondary absorption only present when BTZ-043 is taken together with food. It is unknown if M2 is also converted back into BTZ-043 *in vivo*, hence the BTZ-043 clearance to M2 was modelled as the net result of the BTZ-043-to-M2 conversion.

A double-peak phenomenon was observed for BTZ-043, M1 and M2 individual concentration–time profiles. A first-order secondary delayed absorption into the BTZ-043 central compartment was identified when the drug was taken together with food, using a lag-time model. The secondary delayed absorption was not identifiable when BTZ-043 was taken without food. More complex semiphysiological enterohepatic recirculation models did not significantly improve the model fit. In total, 36% (95% CI: 32%–39%) of the total bioavailable dose was estimated to be absorbed through the secondary delayed absorption route after a typical delay of 1.8 h (95% CI: 1.8–1.9). A schematic representation of the PK model structure is presented in Figure 1.

BTZ-043 administration after standard food was selected as reference to specify the changes in bioavailability of different food types. High-fat food significantly increased the bioavailability of BTZ-043 by 41% (95% CI: 5.0%–78%). When BTZ-043 was taken prior to the standard food, the bioavailability was 27% (95% CI: 12%–42%) lower, and when taken without food, the bioavailability was decreased by 54% (95% CI: 43%–65%). An apparent plateau in bioavailability was observed for doses over 1250 mg daily. No further decrease in the bioavailability was detected between the 1500 and 1750 mg doses (dOFV = −3.3, df = 1). The mean transit time decreased by 64% (95% CI: 53%–72%) when taken together with any kind of food. Furthermore, the relative fraction metabolized into M1 increased by 39% (95% CI: 34%–44%) when BTZ-043 was administered together with food compared with when BTZ-043 was administered without food. The clearance of M2 decreased by 27% (95% CI: 24%–29%) after 10 days on BTZ-043 treatment. The effect was implemented as a dichotomous step after 10 days as a linear model did not improve the model fit and extrapolation after the studied period is uncertain. BTZ-043 clearance was estimated to be 24% (95% CI: 12%–35%) slower in Cape-coloured participants. No effects of age or sex were identified.

**Figure 2.**
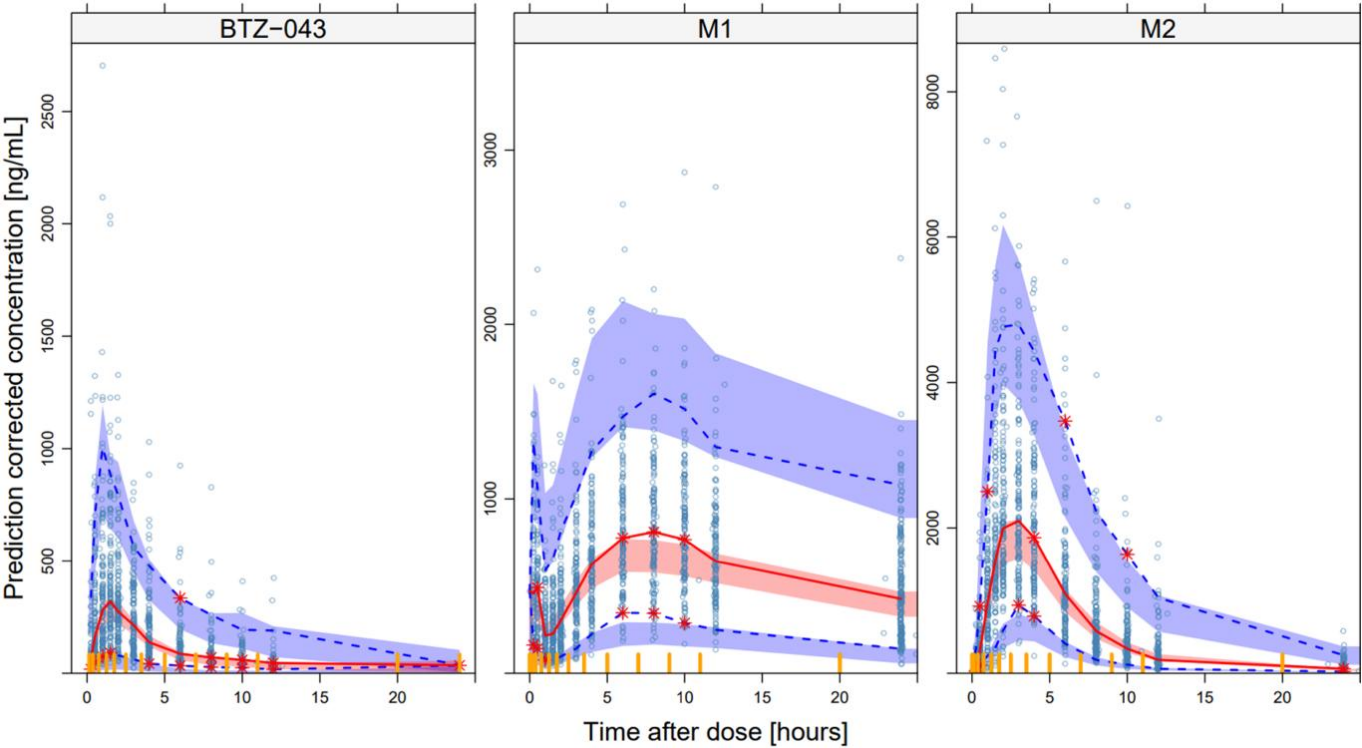
Prediction-corrected VPC showing the observed 2.5th, 50th and 97.5th percentiles (lines) and corresponding 95% CIs from the final PK model. From left to right: predicted BTZ-043, M1 and M2 concentrations over time after dose.

Parameter estimates of the final model are presented in Table 2. A VPC of the final PK model is presented in Figure 2. VPCs stratified on food type are presented in Figures S1–S3. The developed PK model generally described the BTZ-043 PK well. The model-predicted median and 95% CI generally overlapped well with the observed data. Although the model underestimated peak concentrations of M1 in the total dataset, this discrepancy was not as apparent when considering different food types (Figure S2). Furthermore, M2 peak concentrations were slightly underpredicted. This might be due to the complex BTZ-043 metabolism, food and dose effects. The misfit for the final elimination phase of BTZ-043 is likely due to a high percentage of BLQ observations. VPCs on a log scale are presented in Figure S4, and VPCs per dose group in Stage 2 following a BTZ-043 dose 30 min after food are presented in Figure S5.

**Table 2.** Estimated PK model parameters for BTZ-043, M1 and M2

Parameter	Estimate (95% CI SIR)	Variability (CV%) (95% CI SIR)
Structural parameters		
CL/F (L/h)	404 (327–479)	27.2 (21.0–35.4)
*V*/F (L)	764 (303–953)	43.3 (33.3–55.8)
*k*_a_ (h^−1^)	1.69 (1.35–2.21)	128 (97.6–173)
F (%)	100 FIX	IOV = 23.6 (20.0–27.4)
		IIV = 26.5 (19.3–35.7)
MTT (h)	0.340 (0.270–0.419)	92.8 (76.3–116)
Fraction parallel absorption with food (%)	0.644 (0.606–0.679)	
Lag time parallel absorption with food (h)	1.83 (1.76–1.90)	
Q_BTZ-043_/F (L/h)	68.0 (55.0–81.3)	
*V*_pBTZ-043_/F (L)	382 (310–449)	
CL_M1_/(F·fm_M1_) (L/h)	36.0 (29.3–43.2)	43.6 (33.2–56.9)
*V*_M1_/(F·fm_M1_) (L)	894 (699–1100)	48.6 (39.0–60.8)
CL_M2_/(F·fm_M2_) (L/h)	45.8 (37.8–54.4)	30.3 (24.6–36.0)
*V*_M2_/(F·fm_M2_) (L)	19.0 (13.4–25.8)	108 (73.3–180)
Q_M2_/(F·fm_M2_) (L/h)	64.2 (38.6–99.5)	
*V*_pM2_/(F·fm_M2_) (L)	26.8 (21.4–33.2)	
Correlation MTT–*k*_a_ (%)	44.4 (27.8–54.9)	
Covariates		
Dose (>1250 mg) on F (%)	71.0 (51.0–98.0)	
High-fat food on F (%)	141 (105–178)	
Dose prior to standard food on F (%)	72.7 (58.3–88.3)	
No food on F (%)	45.8 (35.2–56.9)	
Dose prior to standard food/no food on MTT (%)	36.0 (27.6–46.8)	
Administration with food on F_M1_ (%)	139 (134–144)	
Cape-coloured race on CL/F (%)^a^	76.2 (65.4–88.5)	
Time effect on CL_M2_/(F·fm_M2_) (%)^b^	73.4 (70.8–76.1)	
Residual error		
Proportional error BTZ-043 (CV%)	50.5 (47.7–53.4)	
Proportional error M1 (CV%)	33.8 (32.4–35.1)	
Proportional error M2 (CV%)	29.0 (28.0–30.1)	

Fm, fraction metabolized; MTT, mean transit time; Q, intercompartmental clearance. CV% was calculated as $\sqrt{(e^{OM^{2}}}-1)$. Disposition parameters were allometrically scaled using a reference total body weight of 70 kg.

^a^Parameterized as relative percentage in Cape-coloured participants versus other participants.

^b^Parameterized as percentage of clearance after Day 10 compared with before Day 10.

**Figure 3.**
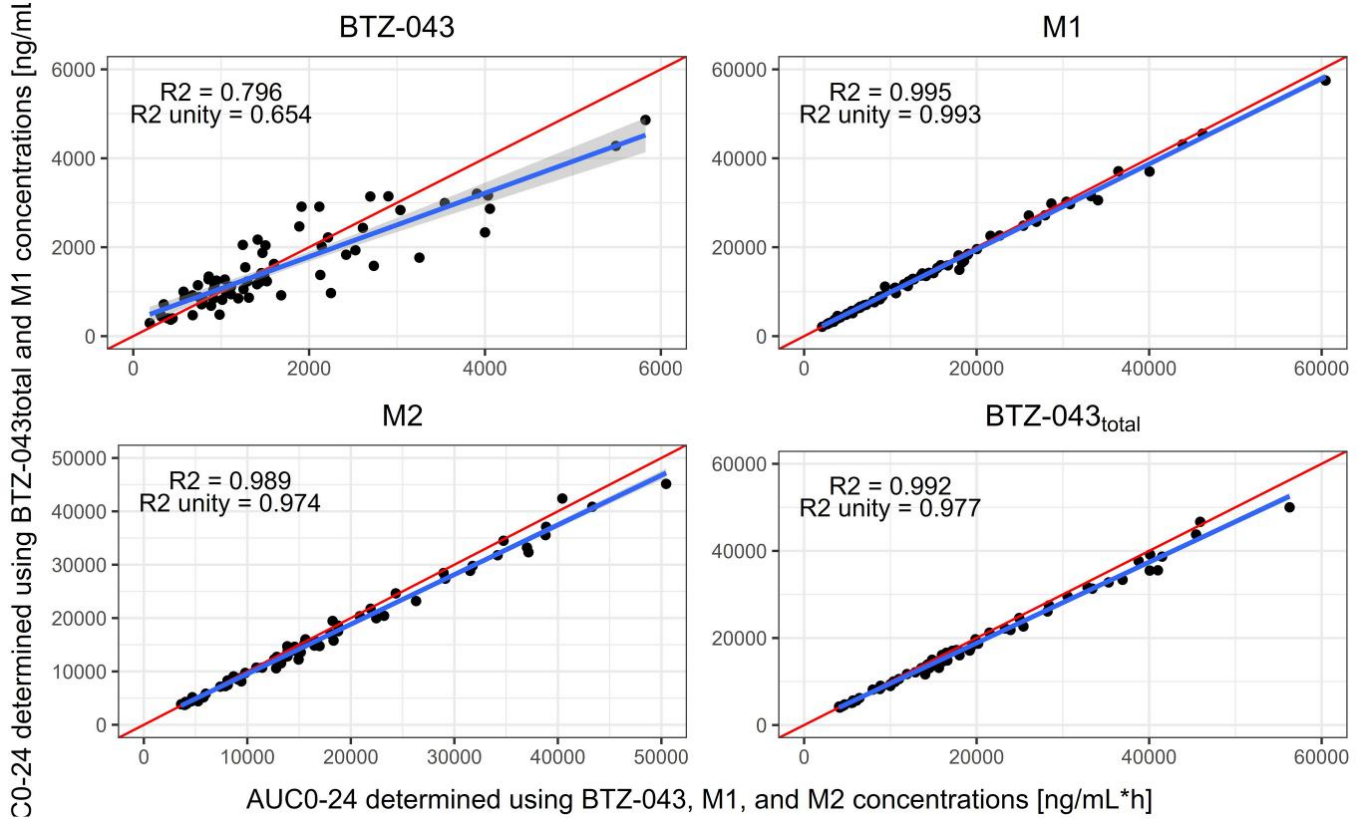
From top left to bottom right: model-predicted AUC_0–24_ of BTZ-043, M1, M2 and BTZ-043_total_ with all analytes as input of the PK model compared with the model-predicted AUC_0–24_ with BTZ-043_total_ and M1 concentrations as input of the PK model. The red line represents the line of unity. The blue line represents the linear regression and the grey shaded area the 95% CI of the linear regression.

Using BTZ-043_total_ and M1 concentrations as input for the PK model, the AUC_0–24_ of each analyte highly correlated with to the predicted AUC_0–24_ of the PK model informed by BTZ-043, M1 and M2 concentrations. Figure 3 shows the correlations between these two methods. M1, M2 and BTZ-043_total_ exposure were best correlated and BTZ-043 exposure showed a more substantial deviation.

### PD model

In total, 921 cfu and 1113 TTP measurements were available for analysis. Of these, 7 cfu and 2 TTP observations were culture negative (cfu < 1.0 cfu/mL or TTP > 25 days). The proportion of observations that were culture negative at baseline (defined as the first 2 days on treatment) was 0.9% for cfu and 0.0% for TTP.

A bilinear model on log scale with estimated node parameter provided the best model fit for both cfu and TTP. The combined model for cfu and TTP was characterized by a shared node parameter, correlated IIV for intercepts and slopes, and a correlated residual error model. The node parameter was estimated at 2 days after the start of treatment. IIV was identified on the baseline bacterial load, the first slope for TTP and the second slopes for cfu and TTP. The model was unable to determine IIV on the first cfu slope. A higher baseline bacterial load correlated with a faster decrease in bacterial load during the first 2 days after the start of treatment for TTP. Before the exposure–response analysis, the node parameter was fixed to the best estimate (48 h) to stabilize the estimation properties of the model. The residual error model was additive on a log scale. A correlation between cfu and TTP observations from sputum samples at the same timepoint was implemented, as well as a replicate error for duplicate cfu and TTP samples. No effect of age, weight and study site was identified.

BTZ-043_total_ AUC_0–24_ and the M2 AUC_0–24_ were identified as the most significant drivers of the decrease in bacterial load during the first 2 days of treatment (dOFV = −12.1 and −13.1, respectively). No exposure–response effect was identified with M1 as driver of the effect. As the BTZ-043_total_ AUC_0–24_ is easier to quantify in future studies, the exposure–response model using this parameter as driver was selected. The first slope of the bacterial load decrease was parameterized as an E_max_ model driven by the BTZ-043_total_ AUC_0–24_ with an estimated EC_50_ at 16 900 ng/mL*h (95% CI: 5510–44 300), similar to the exposure obtained after a 500 mg BTZ-043 dose following a standard breakfast. No exposure–response relationship could be identified over Days 2–14 after the start of treatment.

**Figure 4.**
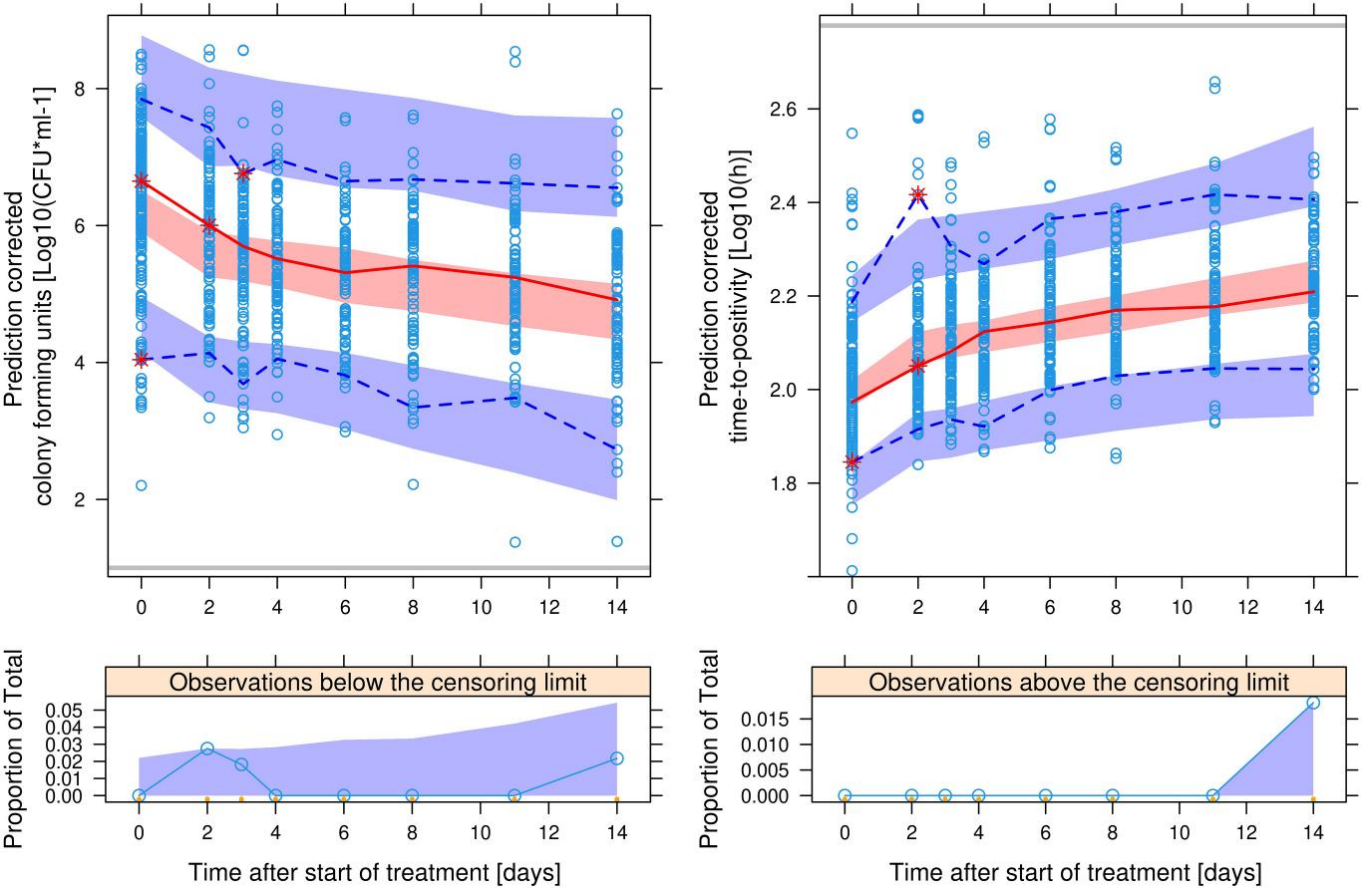
Prediction-corrected VPC showing the observed 2.5th, 50th and 97.5th percentiles (lines) and corresponding 95% CIs from the combined cfu and TTP PD model. Left: predicted cfu over time after the start of treatment, and percentage of predicted and observed censored observations in the lower panel. Right: predicted TTP over time after the start of treatment, and percentage of predicted and observed censored observations in the lower panel. The grey horizontal lines represent the censoring limits.

The PK/PD model described the decrease and variability of bacterial load over time well. A slight underprediction of the baseline bacterial load for cfu was observed. Final PD model parameters are presented in Table 3 and a VPC of the final model is presented in Figure 4.

**Table 3.** Estimated PD model parameters

	Best estimate (SIR 95% CI)	Variability (CV%) (SIR 95% CI)
Fixed effects		
Baseline cfu (log_10_ cfu/mL)	6.20 (5.94–6.42)	15.2 (12.8–17.9)
Baseline TTP (log_10_h)	1.99 (1.97–2.02)	5.14 (4.44–5.91)
E_max_ slope 1 cfu (log_10_ cfu/mL/h)	0.0270 (0.0172–0.0433)	
E_max_ slope 1 TTP (−log_10_h/h)	0.00386 (0.00249–0.00626)	75.9 (35.4–67.1)
Slope 2 cfu (log_10_ cfu/mL/h)	0.00254 (0.00191–0.00311)	75.9 (51.8–117)
Slope 2 TTP (−log_10_h/h)	0.000440 (0.000351–0.000510)	49.7 (34.8–73.3)
Node (h)	48 FIX	
Baseline chance negative culture cfu (%)	0.90	
Baseline chance negative culture TTP (%)	0.00	
EC_50_ BTZ-043_total_ exposure (ng/mL*h)	16 900 (5510–44 300)	
Correlations		
Correlation baseline cfu–baseline TTP (%)	−68.3 (−57.1 to −81.0)	
Correlation intercept cfu–slope 1 TTP (%)	−29.9 (−4.42 to −51.1)	
Correlation intercept TTP–slope 1 TTP (%)	−27.8 (−0.900 to −51.1)	
Correlation slope 2 cfu–slope 2 TTP (%)	77.9 (92.3–56.4)	
Residual error		
Additive error cfu (log_10_ cfu/mL)^a^	0.558 (0.526–0.592)	
Additive error TTP (log_10_h)^a^	0.0624 (0.0597–0.0665)	
Correlation additive error cfu–additive error TTP (%)	−53.3 (−47.0–−59.2)	
Correlation cfu replicate error (%)	89.6 (88.5–91.2)	
Correlation TTP replicate error (%)	85.2 (84.4–86.8)	

CV% was calculated as $\sqrt{(e^{OM^{2}}}-1)$.

^a^The average additive errors and correlations between cfu and TTP replicates is reported here. Parameter estimates for the replicates are presented in the model code shown in the Supplementary material.

response

## Discussion

We characterized the population PK and PD of BTZ-043 and its main metabolites in trial participants diseased with drug-susceptible pulmonary TB, treated with monotherapy over 14 days. An exposure–response relationship was identified during the first 2 days on treatment, which was driven by the model-predicted AUC_0–24_ of the BTZ-043_total,_ i.e. BTZ-043 and metabolite M2. No exposure–response relationship was identified on the second slope (Days 2–14). Participants in the highest dose group in Stage 2 (1000 mg) had on average a 2-fold faster decrease in mycobacterial load compared with participants in the lowest dose group (250 mg) during the first 2 days on treatment.

An apparent plateau in bioavailability was observed for doses of >1250 mg daily when administered without food. Further increase of the dose lead to a less-than-linear increase in exposure to BTZ-043, possibly due to saturable absorption. Administering BTZ-043 alongside food significantly enhances its bioavailability, emphasizing the importance of coadministration with food, and the timing of the dose relative to the food. No plateau in bioavailability was observed within the investigated dose range (250–1000 mg) when administered with food or high-fat food. The absorption process was highly variable between and within participants. A double peak during the absorption was frequently observed, for which the physiological reason remains unclear. An explanation for this effect might be that BTZ-043 could undergo enterohepatic recirculation. However, a semimechanistic model incorporating enterohepatic recirculation did not provide a significantly better model fit compared with the parallel absorption. Emptying of the gallbladder, initiating the recirculation, is triggered by food intake.^22^ This might explain why parallel absorption is only identifiable when BTZ-043 is taken together with food. In ongoing and future planned trials BTZ-043 is given with food.

A decrease in M2 clearance over time on treatment was observed, possibly reflecting a change in enzymatic activity as a result of BTZ-043 administration. When administered together with food, an increase in the fraction metabolized into M1 was identified. As coadministration with food is not expected to have a large effect on systemic clearance/metabolism, we putatively attributed this effect to pre-systemic metabolism of BTZ-043 to M1, which might be higher due to prolonged intestinal passage time when taken together with food. The BTZ-043 clearance was decreased by approximately 24% in participants who identified as being of the ‘Cape-coloured’ race that is common in the South-Western part of South Africa. A potential hypothesis might be that the effect is due to a genetic polymorphism in a BTZ-043-metabolizing enzyme. This effect should be confirmed in a larger population. A limitation of the PK model is that it does not consider BLQ observations, due to model instability using the M3 method. The percentage of BLQ observations was generally underpredicted in the late-elimination phase for BTZ-043.

The bacterial load decreased 5–6-fold faster in the first 2 days on treatment compared with Days 2–14, which is consistent with previous studies of many TB drugs (e.g. isoniazid and isoniazid-containing regimens).^23^ The PK/PD model detected a significant exposure–response relationship during the first 2 days on treatment. It is possible that the study lacked power to identify an exposure–response relationship during Days 2–14, due to the lower absolute decrease in the bacterial load over this period. Alternatively, the exposure to BTZ-043 might already exhibit near-maximal effect during this time period. The fast initial decrease in bacterial load might be explained by the mechanism of action of BTZ-043, inhibition of cell-wall synthesis. Higher doses of BTZ-043 might therefore affect the fast-replicating bacterial subpopulation more than bacteria that are less metabolically active. Several studies have shown that determination of a significant EBA does not necessarily lead to efficacy in long-term clinical trials, or to improved clinical outcomes.^24,25^ Moreover, EBA as determined over 14 days does not necessarily correlate with sterilizing activity, nor does it indicate added efficacy when given together in a regimen instead of using monotherapy. It is hypothesized that EBA studies mainly show the activity against rapidly reproducing bacteria in the lung wall, and not the cavity-penetrating potential or the activity against slow-replicating bacteria. Linezolid and pyrazinamide, both essential sterilizing drugs, also showed a low activity between Days 2 and 7 in Phase 2 trials.^26,27^ Therefore, the choice of the most active dose should ideally be selected based on longer-term efficacy information.

No significant difference in the exposure–response relationship was detected when comparing BTZ-043_total_ or M2 as driver of the antimicrobial effect. This might be due to the high correlation between the BTZ-043_total_ and M2 exposure. BTZ-043_total_ was selected as the effect driver of the antimicrobial effect, considering M2 cannot be directly measured. No exposure–response effect driven by the M1 metabolite was identified. This is consistent with preclinical data that showed a factor 500 lower bactericidal activity for M1 *in vitro* compared with BTZ-043.^3^

Phase 2b-3 trials are needed to assess the added activity of BTZ-043 in a combination regimen and to evaluate longer-term endpoints. In the DECISION trial (NCT05926466) daily doses of 500, 1000 and 1500 mg are being administered over 4 months in combination with bedaquiline and delamanid to assess the decrease in bacterial load (as determined by the TTP in liquid medium) and select the optimal dose of BTZ-043. Our results support the doses selected in these trials, as the EC_50_ of the exposure–response in our study is similar to exposure in the lowest 500 mg dose group.

In conclusion, a PK/PD model of BTZ-043 and its main metabolites was developed. These population PK and PK/PD models are valuable tools for further clinical trial design and analysis with BTZ-043. The wide range of doses studied in this innovative two-stage trial allowed for characterization of the exposure–response relationship. An exposure–response relationship was identified for the first 2 days on treatment, driven by the combined BTZ-043 and M2 exposure.

## Supplementary Material

dkaf076_Supplementary_Data

## Transparency declarations

S.E.K., N.H., V.R.d.J., J.D., P.P.J.P., P.G.D., R.D., K.N., L.M.W., T.D.M., L.H.M.t.B., A.H.D., R.E.A., M.H. and E.M.S. declare receiving grant funding for this work from EDCTP, BMBF, DZIF and Bavarian Ministry to their respective institutions. S.E.K., N.H., J.D., P.P.J.P., L.M.W., T.D.M., L.H.M.t.B., R.E.A., M.H. and E.M.S. declare receiving funding from LigaChem Biosciences for the evaluation of delpazolid to their institutions. E.M.S. declares receiving funding to her institution from Janssen Pharmaceuticals for a scientific project related to bedaquiline, and from TB Alliance for projects related to paediatric development of pretomanid.

## Supplementary data

Figures S1–S5 are available as Supplementary data at *JAC* Online.
