# Supplementary material for: Population pharmacokinetics and exposure–response relationship of the antituberculosis drug BTZ-043
Source: J Antimicrob Chemother. 2025 Mar 28;80(5):1315–23. doi: 10.1093/jac/dkaf076 (PMC12046392; doi:10.1093/jac/dkaf076)
Supplement: dkaf076_Supplementary_Data [file dkaf076_supplementary_data.docx]

**Population pharmacokinetics and exposure-response of the anti-tuberculosis drug BTZ-043**

Simon E KOELE^1*^, Norbert HEINRICH^2,3,4^, Veronique R DE JAGER^5^, Julia DREISBACH^2,3^, Patrick PJ PHILLIPS^6^, Petra GROSS-DEMEL^2,3^, Rodney DAWSON^7^, Kim NARUNSKYy^7^, Leticia M WILDNER^8^, Timothy D MCHUGH^8^, Lindsey HM TE BRAKE^1^, Andreas H DIACON^5^, Rob E AARNOUTSE^1^, Michael HOELSCHER^2,3,4,9^, Elin M SVENSSON^1,10^

^1^ Department of Pharmacy, Radboud Institute for Medical Innovation, Radboud university medical center, Nijmegen, the Netherlands

^2^ Institute of Infectious Diseases and Tropical Medicine, LMU University Hospital, LMU, Munich, Germany

^3^ German Center for Infection Research (DZIF), Munich Partner Site, Munich, Germany

^4^ Fraunhofer Institute for Translational Medicine and Pharmacology ITMP, Immunology, Infection and Pandemic Research, Munich, Germany

^5^ TASK, Cape Town, South Africa

^6^ UCSF Center for Tuberculosis, University of California San Francisco, San Francisco, United States

^7^ University of Cape Town Lung Institute, Cape Town, South Africa

^8^ UCL Centre for Clinical Microbiology, University College London, London, UK

^9^ Unit Global Health, Helmholtz Zentrum München, German Research Center for Environmental Health (HMGU), Neuherberg, Germany

^10^ Department of Pharmacy, Uppsala University, Uppsala, Sweden

**Overview of content:**

Figure S1: VPC of BTZ-043 stratified on food-type Page 2

Figure S2: VPC of M1 stratified on food-type Page 2

Figure S3: VPC of M2 stratified on food-type Page 3

Figure S4: VPC on log-scale Page 3

Figure S5: VPC stage 2 per dose Page 4

Pharmacokinetic model code Page 5

Pharmacodynamic model code Page 9

Bioanalysis methods Page 12


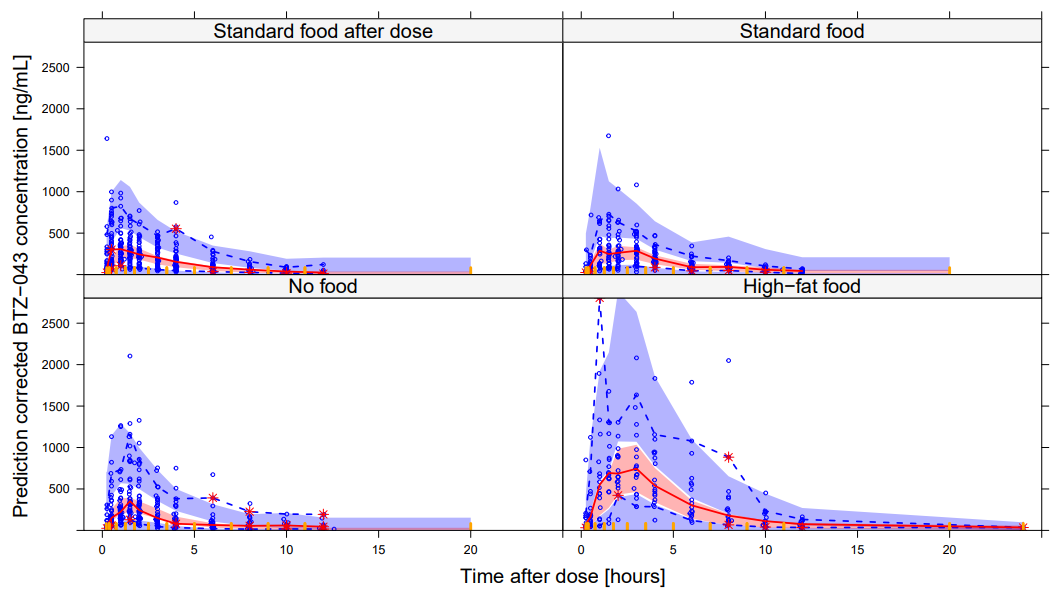


*Figure S1: Prediction corrected VPC showing the observed 2.5^th^, 50^th^, and 97.5^th^ percentiles (lines) and the 95% confidence intervals for the same percentiles from the PK model. From top left to bottom right: Predicted BTZ-043 concentrations for standard food after dose, standard food, no food, and high-fat food.*


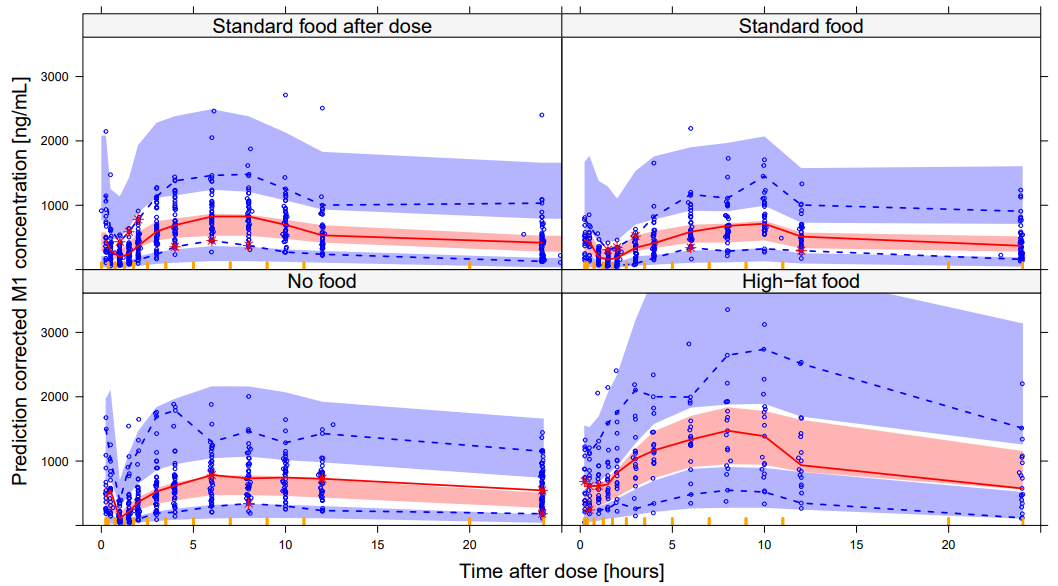


*Figure S2: Prediction corrected VPC showing the observed 2.5^th^, 50^th^, and 97.5^th^ percentiles (lines) and the 95% confidence intervals for the same percentiles from the PK model. From top left to bottom right: Predicted M1 concentrations for standard food after dose, standard food, no food, and high-fat food.*


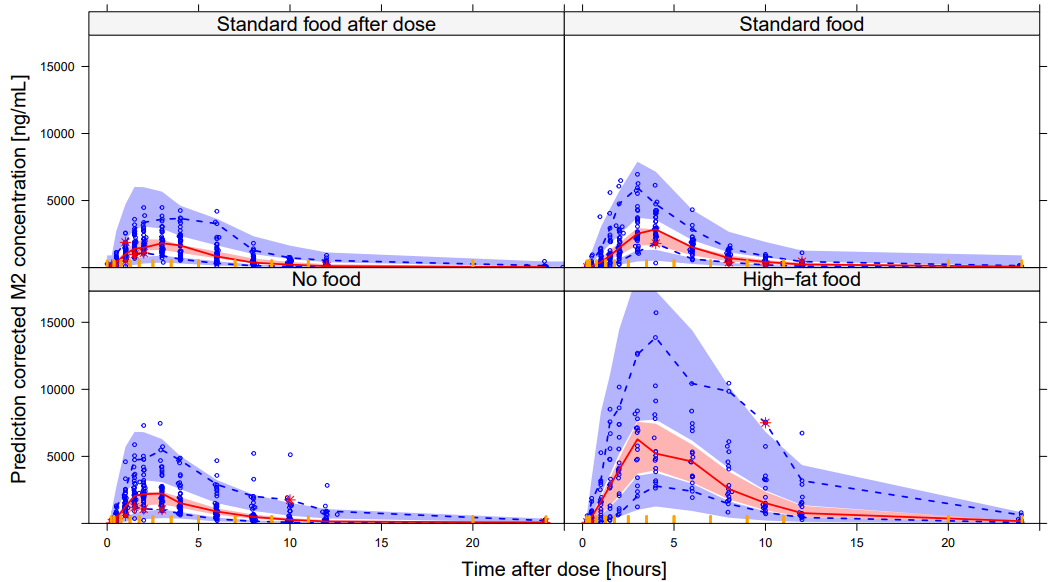


*Figure S3: Prediction corrected VPC showing the observed 2.5^th^, 50^th^, and 97.5^th^ percentiles (lines) and the 95% confidence intervals for the same percentiles from the PK model. From top left to bottom right: Predicted M2 concentrations for standard food after dose, standard food, no food, and high-fat food.*

*
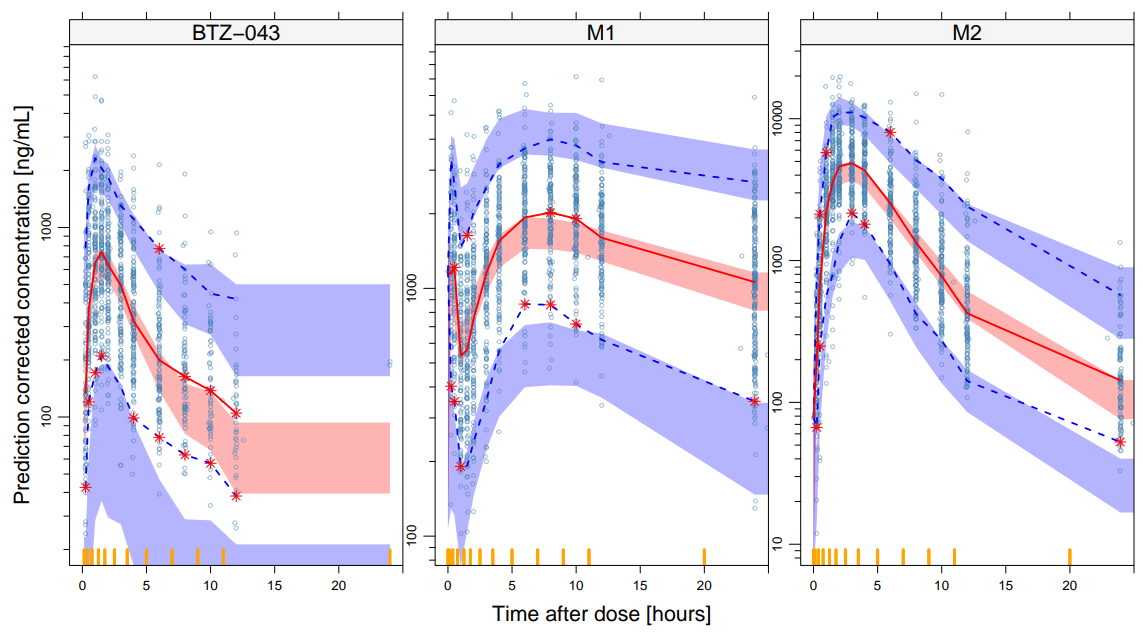
*

*Figure S4: Prediction corrected VPC showing the observed 2.5^th^, 50^th^, and 97.5^th^ percentiles (lines) and corresponding 95% confidence intervals from the final PK model on log-scale. From left to right: Predicted BTZ-043, M1, and M2 concentrations over time after dose.*

*
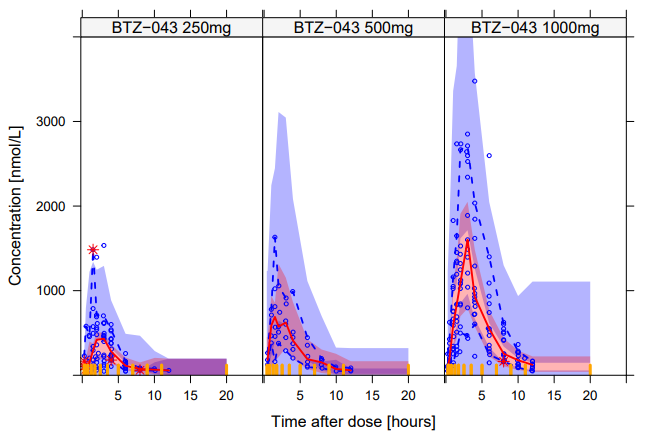
*

*
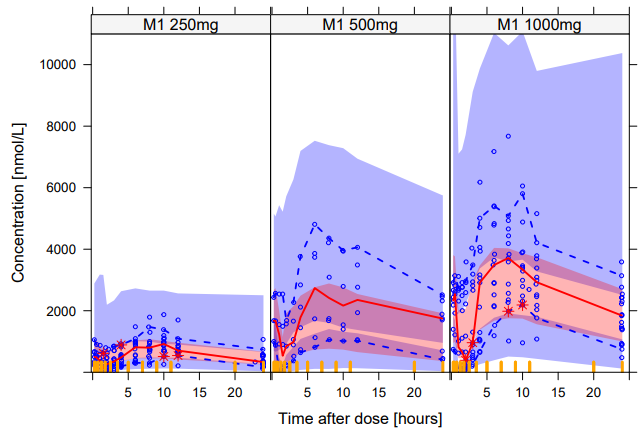
*

*
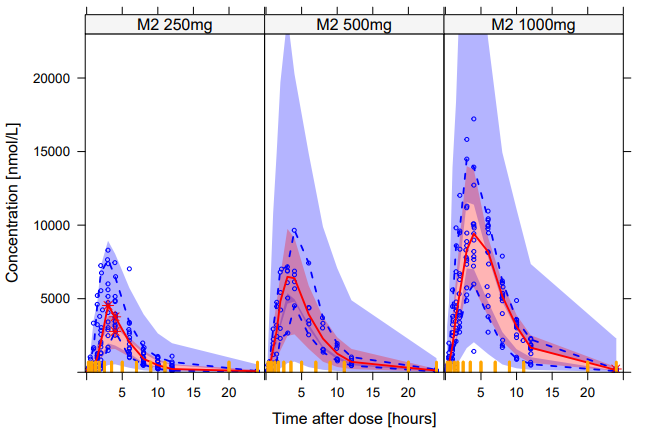
*

*Figure S5: Uncorrected VPCs showing the observed 2.5^th^, 50^th^, and 97.5^th^ percentiles (lines) and corresponding 95% confidence intervals from the final PK model for participants in Stage 2 receiving BTZ-043 30 minutes after the start of intake of a standard breakfast stratified per dose. From top to bottom: Predicted BTZ-043, M1, and M2 concentrations over time after dose. From left to right: 250mg, 500mg, and 1000mg of BTZ-043.*

**Pharmacokinetic model code:**

$PROBLEM PK BTZ043

$INPUT ID DOSE DAY TIME CMT EVID AMT FLAG DV DOSEX FOOD RACE WT

$DATA ….csv IGNORE=@

$SUBROUTINE ADVAN13 TOL=6

$MODEL NCOMP=9 COMP=(DEPOT) COMP=(DEPOT2) COMP=(BTZ043)

COMP=(TRANS1) COMP=(TRANS2) COMP=(M1) COMP=(M2) COMP=(M2peripheral) COMP=(BTZ043peripheral)

;Data dictionary

;ID = Participant ID

;DOSE = Dose [mg]

;DAY = Day after start of treatment [day]

;TIME = Time after start of treatment [h]

;CMT = Compartment number

;EVID = Event ID

;AMT = Drug amount [mg]

;FLAG = Compound identifier [1=BTZ-043, 2=M1, 3=M2]

;DV = Observed concentration [nM/L]

;DOSEX = Identifier second dose for parallel absorption [0=no, 1=yes]

;FOOD = Type of food administered together with BTZ-043 [0=no food, 1= High fat food, 2=standard food after dose, 3= standard food before dose]

;RACE = Race [1=Cape-colored, 0=other]

;WT = Weight [kg]

$PK

;-----------------------Allometric scaling--------------------------

AlloCL=(WT/70)**0.75

AlloV=(WT/70)**1

;--------------------------Food effects----------------------------

FOOD1 = 0 ; High fat food

IF(FOOD.EQ.1) FOOD1=1

FOOD2 = 0 ; Dose prior to food

IF(FOOD.EQ.2) FOOD2=1

FOOD3= 0 ; Standard food before dose

IF(FOOD.EQ.3) FOOD3=1

WITHFOOD=0

IF(FOOD.EQ.3.OR.FOOD.EQ.1)WITHFOOD=1

;-----------------------Structural parameters----------------------

TVCL = THETA(1)

TVV = THETA(2)

TVKA = THETA(3)

TVMTT = THETA(4)

MTTeff = 1

IF(WITHFOOD.EQ.0)MTTeff = THETA(5)

TVF1 = 1

IF(WITHFOOD.EQ.1)TVF1 = THETA(6)

ALAG2 = THETA(7)

FDose = 1

IF(DOSE.GT.1250)FDose = THETA(8)

F_highfat = 1

IF(FOOD.EQ.1)F_highfat = THETA(9)

F_nofood = 1

IF(FOOD.EQ.0)F_nofood = THETA(10)

F_latefood = 1

IF(FOOD.EQ.2)F_latefood = THETA(11)

errM0 = THETA(12)

TVCLM1 = THETA(13)

TVVM1 = THETA(14)

errM1 = THETA(15)

TVCLM2 = THETA(16)

TVVM2 = THETA(17)

errM2 = THETA(18)

TVQM2 = THETA(19)

TVVpM2 = THETA(20)

CLM2time = THETA(21)

CLeffRace = 1

IF(RACE.EQ.1)CLeffRace = THETA(22)

FM1FOOD = 1

IF(WITHFOOD.EQ.0)FM1FOOD = THETA(23)

TVQM0 = THETA(24)

TVVpM0 = THETA(25)

;---------------------------OCCASION definition---------------------

OCC1 = 0

OCC2 = 0

OCC3 = 0

IF(DAY.EQ.1) OCC1 = 1

IF(DAY.EQ.12.AND.STAGE.EQ.1) OCC2 = 1

IF(DAY.EQ.14) OCC3 = 1

;--------------------------IIV/IOV definition----------------------

IIVCL = ETA(1)

IIVV = ETA(2)

IOVF = ETA(3)*OCC1 + ETA(4)*OCC2 + ETA(5)*OCC3

IOVMTT = ETA(6)*OCC1 + ETA(8)*OCC2 + ETA(10)*OCC3

IOVKA = ETA(7)*OCC1 + ETA(9)*OCC2 + ETA(11)*OCC3

IIVF = ETA(12)

IIVCLM1 = ETA(13)

IIVVM1 = ETA(14)

IIVCLM2 = ETA(15)

IIVVM2 = ETA(16)

;-----------------------------PK parameters-------------------------

;M0

KA = TVKA* EXP(IOVKA)

KTR = 1/(TVMTT*EXP(IOVMTT)*MTTeff)

CL = TVCL * EXP(IIVCL) *AlloCL * CLeffRace

V = TVV * EXP(IIVV) *AlloV

QM0 = TVQM0 *AlloCL

VpM0 = TVVpM0 *AlloV

;M1

CLM1 = TVCLM1/FM1FOOD *EXP(IIVCLM1)*AlloCL

VM1 = TVVM1/FM1FOOD *EXP(IIVVM1) *AlloV

;M2

CLM2timeEff=1

IF(DAY.GT.10)CLM2timeEff=1*CLM2time

CLM2 = TVCLM2 *EXP(IIVCLM2)*AlloCL *CLM2timeEff

VM2 = TVVM2 *EXP(IIVVM2) *AlloV

QM2 = TVQM2 *AlloCL

VpM2 = TVVpM2 *AlloV

;---------------------------Bioavailability-------------------------

F1 = TVF1 *1000000/431.39 *EXP(IOVF) *EXP(IIVF) *F_highfat *F_nofood *F_latefood *FDose ; DV in nmol/L. AMT in mg. Mw = 431.39 g/mol. To get dose in nmol = 10^6/431.39

F2 = (1- TVF1) *1000000/431.39 *EXP(IOVF) *EXP(IIVF) *F_highfat *F_nofood *F_latefood *FDose

$DES

DADT(1) = -KTR*A(1)

DADT(2) = -KA*A(2)

DADT(3) = KA*A(5) + KA*A(2) - CL/V*A(3) - QM0/V*A(3)+ QM0/VpM0*A(9)

DADT(4) = KTR*A(1) - KTR*A(4)

DADT(5) = KTR*A(4) - KA*A(5)

DADT(6) = CL/V*A(3) - CLM1/VM1*A(6)

DADT(7) = CL/V*A(3) - CLM2/VM2*A(7)- QM2/VM2*A(7) + QM2/VpM2*A(8)

DADT(8) = QM2/VM2*A(7) - QM2/VpM2*A(8)

DADT(9) = QM0/V*A(3) -QM0/VpM0*A(9)

$ERROR

IF(FLAG.EQ.1)IPRED = A(3)/V

IF(FLAG.EQ.2)IPRED = A(6)/VM1

IF(FLAG.EQ.3)IPRED = A(7)/VM2

W=1

IF(IPRED.NE.0.AND.FLAG.EQ.1) W = SQRT(errM0**2*IPRED**2)

IF(IPRED.NE.0.AND.FLAG.EQ.2) W = SQRT(errM1**2*IPRED**2)

IF(IPRED.NE.0.AND.FLAG.EQ.3) W = SQRT(errM2**2*IPRED**2)

IRES = DV-IPRED

IWRES = IRES/W

Y=IPRED + ERR(1)*W

$THETA

(0, 404) ; 1 CL

(0, 764) ; 2 V

(0, 1.69) ; 3 Ka

(0, 0.34) ; 4 MTT

(0, 0.36) ; 5 late food MTT

(0, 0.644,0.9999) ; 6 F1 w food

(0, 1.83) ; 7 lagtime abs 2

(0, 0.71) ; 8 Fhighdose

(0, 1.41) ; 9 F highfat

(0, 0.458) ; 10 F nofood

(0, 0.727) ; 11 F late food

(0, 0.477) ; 12 prop err M0

(0, 36) ; 13 CLM1

(0, 894) ; 14 VM1

(0, 0.329) ; 15 prop err M1

(0, 45.8) ; 16 CLM2

(0, 19) ; 17 VM2

(0, 0.285) ; 18 prop err M2

(0, 64.2) ; 19 QM2

(0, 26.8) ; 20 VpM2

(0, 0.734) ; 21 CLM2timeEff

(0, 0.762) ; 22 CLeffRace

(0, 1.39) ; 23 FM1food

(0, 68) ; 24 QM0

(0, 382) ; 25 VpM0

$OMEGA

0.0715 ; 1 IIV CL

0.172 ; 2 IIV V

$OMEGA BLOCK(1)

0.0543 ; 3 IOV F

$OMEGA BLOCK(1) SAME

$OMEGA BLOCK(1) SAME

$OMEGA BLOCK(2)

0.621 ; 6 IIV MTT

0.345 0.972 ; 7 IOV KA

$OMEGA BLOCK(2) SAME

$OMEGA BLOCK(2) SAME

$OMEGA

0.0681 ; 12 IIV F

0.174 ; 13 IIV CLM1

0.212 ; 14 IIV VM1

0.0878 ; 15 IIV CLM2

0.77 ; 16 IIV VM2

$SIGMA 1 FIX

$ESTIMATION METHOD=1 INTER MAXEVAL=9999 NOABORT SIGL=6 NSIG=2 PRINT=1

$COVARIANCE UNCONDITIONAL PRINT=E

**Pharmacodynamic model code:**

$PROB BTZ-043 pharmacodynamic model CFU and TTP observations

$INPUT ID TIME DV FLAG BLQ EVID MDV REP L2 EXMET

$DATA ….csv IGNORE =@

;Data dictionary

;ID =Subject ID

;TIME =Time after start of treatment (h)

;DV =Bacterial load (log10(CFU*ml-1),log10(TTP))

;FLAG =Biomarker (1=CFU, 2=TTP)

;BLQ =Below limit of quantification (1=yes, 2=no)

;EVID =Event ID

;MDV =Missing dependent variable

;REP =CFU or TTP replicate (1 or 2)

;L2 =L2 data item

;EXMET =Exposure metric

$PRED

;----------------------------- PD model-----------------------------

INTERCEPTCFU = THETA(1) *EXP(ETA(1))

INTERCEPTTTP = THETA(2) *EXP(ETA(2))

BETA1BTZCFU = (EXMET * THETA(3))/(THETA(8)+(EXMET))

BETA2BTZCFU = THETA(4) *EXP(ETA(4))

BETA1BTZTTP = (EXMET * THETA(5))/(THETA(8)+(EXMET))

BETA2BTZTTP = THETA(6) *EXP(ETA(5))

NODE = THETA(7)

IF(FLAG.EQ.1)THEN

BETA1=BETA1BTZCFU

BETA2=BETA2BTZCFU

ELSE

BETA1=BETA1BTZTTP

BETA2=BETA2BTZTTP

ENDIF

CFU1= INTERCEPTCFU - BETA1*TIME

CFUatNODE= INTERCEPTCFU - BETA1*NODE

CFU2= CFUatNODE - BETA2*(TIME-NODE)

TTP1= INTERCEPTTTP + BETA1*TIME

TTPatNODE= INTERCEPTTTP + BETA1*NODE

TTP2= TTPatNODE + BETA2*(TIME-NODE)

CFU = CFU1

IF (TIME.GT.NODE) CFU= CFU2

TTP = TTP1

IF (TIME.GT.NODE) TTP= TTP2

IF(FLAG.EQ.1) IPRED = CFU

IF(FLAG.EQ.2) IPRED = TTP

;----------- PROBABILITY OF BACTERIAL PRESENCE ---------------------

;Change of negative culture result during first three days on treatment

IF (FLAG.EQ.1) PBAC = 0.009 ;CFU

IF (FLAG.EQ.2) PBAC = 0 ;TTP

;Error model

IF (FLAG.EQ.1.AND.REP.EQ.1) ADDERR = EPS(1)

IF (FLAG.EQ.2.AND.REP.EQ.1) ADDERR = EPS(3)

IF (FLAG.EQ.1.AND.REP.EQ.2) ADDERR = EPS(2)

IF (FLAG.EQ.2.AND.REP.EQ.2) ADDERR = EPS(4)

;----------------------------M3 code--------------------------------

IF (FLAG.EQ.1) SD = SQRT(SIGMA(1,1))

IF (FLAG.EQ.2) SD = SQRT(SIGMA(3,3))

LLOQ=1 ;LLOQ for CFU

ULOQ=LOG10(25*24) ;ULOQ for TTP

DUMLLOQ=(LLOQ-IPRED)/SD

DUMULOQ=(IPRED-ULOQ)/SD

IF (FLAG.EQ.1) CUMD=PHI(DUMLLOQ)

IF (FLAG.EQ.2) CUMD=PHI(DUMULOQ)

IF (BLQ.EQ.2) THEN

F_FLAG=0

Y=IPRED + ADDERR

IRES = DV - IPRED

ENDIF

IF (BLQ.EQ.1) THEN

F_FLAG=1

Y=CUMD+PBAC-(CUMD*PBAC)

MDVRES = 1

ENDIF

$THETA

(0, 6.2) ;1 INTERCEPTCFUP1

(0, 1.99) ;2 INTERCEPTTTPP1

(0, 0.027) ;3 B1CFU

(0, 0.00254) ;4 B2CFU

(0, 0.0039) ;5 B1TTP

(0, 0.0004) ;6 B2TTP

(0, 48) FIX ;7 NODE

(0, 16900) ; 8 EXRES

$OMEGA BLOCK(3)

0.0229 ;1 INTECEPTCFU

-0.0053 0.0026 ;2 INTECEPTTTP

-0.0211 -0.0066 0.217 ;3 SLOPE1TTP

$OMEGA BLOCK(2)

0.455;4 SLOPE2CFU

0.247 0.221;5 SLOPE2TTP

$SIGMA BLOCK(4)

0.299 ; ADD error CFU1

0.278 0.322 ; ADD error CFU2

-0.018 -0.0194 0.0043 ; ADD error TTP1

-0.0175 -0.0189 0.0033 0.0035 ; ADD error TTP2

$ESTIMATION METHOD=1 INTERACTION LAPLACIAN NUMERICAL SLOW MAXEVAL=9999 NSIG=2 PRINT=1

$COV UNCONDITIONAL

**Bioanalytical methods BTZ-043**

Quantitative analysis of BTZ-043, M1, and M2 was performed using two separate methods. Method 1 quantified the BTZ-043_total_ (BTZ-043+ M2, converted to BTZ-043), and method 2 quantified the BTZ-043 and M1. M2 concentrations were subsequently determined by subtracting the BTZ-043 measured with method 2 from the BTZ-043_total_ from method 1. Chromatographic parameters and equipment were identical for both methods, only the sample work-up was different. The methods were validated according to the European Medicine Agency’s “Guideline on bioanalytical method validation”.

Both methods were performed using a Triple Quad 5500 Liquid Chromatography with tandem mass spectrometry (LC-MS-MS) consisting of a LC-pump, auto-sampler and column oven. Chromatic separation was carried out using a HSS T3 (50 mm x 2.1 mm, 1,8 um) column (Waters, Etten-Leur, The Netherlands). The autosampler temperature was kept at 4 degrees Celsius. The mobile phase consisted of a gradient with 0.1% formic acid in water and acetonitrile with a flow rate of 0.600 mL/min. The mass spectrometer was used in the positive ion electrospray ionization mode using multiple-reaction monitoring. The Analyst 1.6.2. system was used as controlling software (MDS Sciex, Framingham, USA).

Sample work-up Method 1 (BTZ-043_total_):

Samples were worked up in a 96-wells format. 10.0 µL of each sample was added to a 1000 µL 96-deepwell plate. 20.0 µL AL-IS (analyte-labeled internal standard), 10.0 µL formic acid (50%) and 10.0 µL hemolytic plasma (0.5%) were added to facilitate back- conversion of all M2 to BTZ-043 in order to form BTZ-043_total_. Samples were centrifuged for 1 min at 1500rpm and shaken on a Mix Mate for 5 min at 1500rpm. Precipitation was performed with 800uL acetonitrile. The 96-deepwell plates were sealed, shaken on a Mix Mate for 1 min at 1500rpm and centrifuged for 5 min at 4500rpm. 5.00 µL was injected into the LC-MS system.

The within run accuracy of eight quality control samples measured in six-fold was 98.6% to 101.2% for BTZ-043_total_ and the precision was 0.7% to 5.6%.
The overall intra-run accuracy of five different concentrations measured in six-fold was determined to be 99.1% to 103.0% and the precision was 1.3% to 7.4%.
The inter-run accuracy determined using six sets of QC samples (5 concentrations each) in three different runs was determined to be 99.4% to 102.3%. The precision was 1.8% to 6.2%. The lower limit of quantification was determined at 20.0 ng/mL.
To check if all M2 was back converted to BTZ-043, validation samples containing only the reference compound M2 were prepared. During extraction all the M2 should be converted into BTZ-043. After extraction the BTZ-043 concentrations were determined as described above. The measurements showed that the whole amount of M2 was converted into BTZ-043 (bias -4.5% to -7.3%).

Sample work-up for Method 2 (BTZ-043 and M1):

At the clinical site, 5 µL of an ascorbic acid solution (250mg/mL) were added to 250 µL of plasma to avoid back-conversion of M2 to BTZ-043.

At the analytical laboratory, samples were worked up in a 96-wells format. 10.0 µL of each sample was added to a 1000 µL 96-deepwell plate. 20.0 µL AL-IS and 10.0 µL 250ng/mL ascorbic acid solution was added. Precipitation was performed with 800uL methanol. The 96-deepwell plates were sealed, shaken on a Mix Mate for 1 min at 1500rpm and centrifuged for 5 min at 4500rpm. 5.00 µL was injected into the LC-MS system.

In summary, the within run accuracy of eight quality control samples measured in seven-fold was 96.8% to 101.4% for BTZ-043 and 96.0% to 102.4% for M1. The precision was 1.4% to 4.7% for BTZ-043 and 1.5% to 5.9% for M1.
The overall intra-run accuracy of five different concentrations measured in six-fold was determined to be -98.1% to 104.0% for BTZ-043 and -97.1% to 103.7% for M1. The precision was 1.8% to 6.3% for BTZ-043 and 1.5% to 6.7% for M1.
The inter-run accuracy determined using six sets of QC samples (5 concentrations each) in three different runs was determined to be 97.6% to 100.5% for BTZ-043 and 97.7% to 106.6% for M1. The precision was 2.4% to 10.1% for BTZ-043 and 4.5% to 7.3% for M1. The lower limit of quantification was determined at 20.0 ng/mL for both analytes.
